# A green method to extract rutin from *Sophora japonica* L.

**DOI:** 10.1016/j.mex.2023.102479

**Published:** 2023-11-08

**Authors:** Nhan Trong Le, Trieu Phat Dac Nguyen, Duc Viet Ho, Huong Thanh Phung, Hoai Thi Nguyen

**Affiliations:** a Faculty of Pharmacy, Hue University of Medicine and Pharmacy, Hue University, Hue City, Viet Nam; b Hanoi University of Pharmacy, Ha Noi City, Viet Nam

**Keywords:** A green method to extract rutin, Rutin, Green solvents, Deep eutectic solvents, Extraction

## Abstract

*Sophora japonica* L. contains high levels of rutin, which has great potential for use in pharmaceutical products for the treatment of diseases related to the cardiovascular and circulatory systems. We proposed a method of extracting rutin from *S. japonica* by using a green solvent.

• Green deep eutectic solvents (DESs) of choline chloride and ethylene glycol (ChCl-Eth) showed the highest extraction efficiency of rutin from *S. japonica*.
• Under optimal conditions, the extraction yield of ChCl-Eth was 1.34 times higher than that of methanol as solvent.
• Rutin was recovered from the DES extracts using water as the antisolvent with a high recovery yield, and the DESs of ChCl-Eth could be productively recovered and reused at least 3 times.

## Graphical abstract

**Figure:**
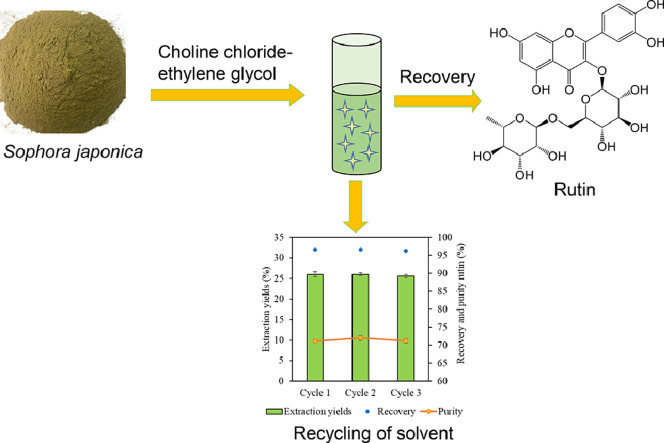

Specifications table

**Table:** 

Subject area:	Chemical Engineering
More specific subject area:	Green extraction
Name of your method:	A green method to extract rutin
Name and reference of original method:	Not applicable
Resource availability:	Standard analytical, laboratory equipment and instruments

## Method details

Deep eutectic solvents (DESs) are composed of cheaper and more environmentally friendly constituents than organic solvents, including hydrogen bond donors (HBDs) such as polyalcohols, carboxylic acids, sugars, amines and a hydrogen bond acceptor (HBA) such as quaternary ammonium salt [1]. Hydrogen bonding formed in DESs is the predominant interaction of HBD and HBA [2]. Until now, DESs have been known as a generation of green solvents and sustainable solutions because of the safety, non-toxicity, low cost and high biodegradability of their components [3].

In recent years, DESs have been studied and applied in different fields; for example, organic synthesis, electrochemistry, extraction, biotechnology and biomedicine [4]. In addition, DESs are also used to improve the solubility of hydrophobic drugs [5], [6], [7], [8], and drug delivery systems [8]. In the extraction and separation of natural compounds of plant origin, several studies have been performed to evaluate the extraction efficiency of phenolics, flavonoids, terpenoids and alkaloids [9], [10], [11], [12]. Therefore, the application of DESs, especially in natural product extraction and general science, is a promising new trend at present and in the future.

## Chemicals and materials

Flower buds powder of *S. japonica* (sieve 0.5–1.0 mm).

Rutin trihydrate (≥94 %, HPLC, Sigma-Aldrich Co., Missouri, US),

Choline chloride (99 %, Thermo Fisher Scientific, Co., Massachusetts, US),

Ethylene glycol (Xilong Scientific Co., Ltd., Guangdong, China).

Methanol (HPLC)

Acetic acid (HPLC)

Acetonitrile (HPLC)

Deionized water

## DES preparation

The DES of ChCl-Eth was obtained by mixing 69.81 g of choline chloride and 93.105 g of ethylene glycol (a molar ratio of 1:3) in a 500 ml conical flask. The mixture of ingredients of DES was constantly stirred at 80 °C for 6 h using heated magnetic stirrers until a homogeneous fluid was obtained. The DES was then cooled down before being stored in a desiccator [13]. Next, 90 g of DES was mixed with 10 g of deionised water to prepare the extraction solvent.

## Rutin extraction

Extraction was performed by mixing 500 mg material with 8.5 mL of the DESs containing 10 % water at temperature of 60 °C for 60 min then followed by a centrifugation (4000 rpm, 10 min) using an Electronic Centrifuge 80–2 (Jiangsu Zhengji Instruments Co., Ltd., China) to separate solids from liquids. Methanol was used as a diluent for the purpose of assaying rutin by HPLC. The liquids were diluted 10 times with methanol and filtered through 0.45 μm polytetrafluoroethylene filters (Whatman plc., Buckinghamshire, United Kingdom). Finally, the rutin content was assayed by HPLC.

## Recovery of rutin from the DESs extract

To recover rutin from the DESs extract, water was used as an antisolvent to recover the target compound from the DESs extract. After rutin was extracted under optimal conditions, 40 mL deionized water was added to 10 mL the DESs extract, and precipitation was performed at 25 °C for 24 h. Then, the precipitate was collected after a centrifugation (4000 rpm, 10 min) and filtered through filter paper. The DESs were recovered from the residual solution after removing the precipitate by evaporating water using a vacuum evaporator (R-300, Buchi Labortechnik, Switzerland). DESs were recovered by adding water to obtain 10 % water containing solutions [14]. The recovered ChCl-Eth was used to extract rutin with fresh biomass.

## Method optimization and validation

### Extraction of rutin

To evaluate the final results of the optimised method for rutin extraction from *S. japonica* using DES, we first investigated the extraction efficiency of 16 DESs with different components (Table 1). The results showed that DESs of ChCl-Eth gave the best rutin extraction efficiency. Thus, ChCl-Eth was selected to optimise the extraction process. The conditions affecting extraction, including the ChCl-Eth ratio, the DES water content, the liquid-solid ratio, the extraction time and the extraction temperature, were optimised. The optimal extraction conditions were as follows: a liquid-solid ratio of 17 ml/g, an extraction time of 60 min, and an extraction temperature of 60 °C. Under these optimal conditions, the maximum yield of rutin was predicted to be 26.21 %. The tested value was determined to be 26.20 ± 0.37 %, which was very close to the predicted value of 26.21 % (*p* > 0.05). Finally, we compared the extraction efficiency of rutin from *S. japonica* using DESs of ChCl-Eth and methanol as extraction solvents. The results showed that the extraction yield with ChCl-Eth was 1.34 times higher than that of methanol (19.49 ± 0.33 %).

**Table 1:** Extraction efficiency of rutin from *S. japonica* with different DESs.

No.	Abbreviation	Combination	Molar ratio	Extraction yields (%)
1	ChCl-Cit	Choline chloride – Citric acid	1 : 1	2.95 ± 0.12
2	ChCl-Lac	Choline chloride – Lactic acid	1 : 2	10.38 ± 0.24
3	ChCl-Ace	Choline chloride – Acetic acid	1 : 2	18.06 ± 0.46
4	ChCl-For	Choline chloride – Formic acid	1 : 2	7.23 ± 0.05
5	ChCl-Oxa	Choline chloride – Oxalic acid	1 : 1	4.58 ± 0.22
6	ChCl-Tar	Choline chloride – Tartaric acid – Water	1 : 1 : 1	2.11 ± 0.15
7	ChCl-Gly	Choline chloride – Glycerol	1 : 2	8.79 ± 0.06
8	ChCl-Pro	Choline chloride – Propylene glycol	1 : 2	16.94 ± 0.35
9	ChCl-Eti	Choline chloride – Ethylene glycol	1 : 2	20.39 ± 0.62
10	ChCl-Sor	Choline chloride – Sorbitol	1 : 1	4.00 ± 0.17
11	ChCl-Glu	Choline chloride – Glucose	1 : 1	1.02 ± 0.06
12	ChCl-Fruc	Choline chloride – Fructose	1 : 1	1.93 ± 0.13
13	ChCl-Xyl	Choline chloride – Xylose – Water	1 : 1 : 1	0.12 ± 0.06
14	ChCl-Suc	Choline chloride – Sucrose – Water	1 : 2 : 2	2.15 ± 0.02
15	ChCl-Mal	Choline chloride – Maltose – Water	1 : 2 : 2	3.19 ± 0.12
16	ChCl-Act	Choline chloride - Acetamide	1 : 2	2.88 ± 0.10

### Recovery of rutin and recycling of the solvent

First, the rutin recovery examination was carried out with various rates of the ChCl-Eth extract and water (1:1, 1:2, 1:3, 1:4 and 1:5, v/v) and the results are presented in Table 2. Recovery yields were calculated as the percentage of rutin that was recovered in the precipitate, in comparison to the amount of rutin initially present in 10 mL of the DES extract. The observed results show that at an amount of water equal to the volume of the extract (ratio of 1:1), rutin was not recovered from the DES extract. The peak recovery (94.86 %) was accomplished with an extract:water ratio of 1:4.

**Table 2:** The recovery efficiency of rutin from the DESs of ChCl-Eth extract.

The DESs extract: water ratios (v/v)	Recovery yields (%)
1 : 1	0
1 : 2	49.59 ± 1.05
1 : 3	91.24 ± 2.73
1 : 4	94.86 ± 3.47
1 : 5	66.90 ± 0.27

Next, the DESs of ChCl-Eth were recovered from the mixture solution of DES extracts and water as an antisolvent. Then, under optimised conditions, the recovered DESs were used in new extraction cycles with fresh *S. japonica* biomass and rutin recovery was performed with an antisolvent. The extraction efficiency, recovery yield and purity of obtained rutin in each cycle were evaluated. As shown in Table 3, the extraction yields (25.57 to 26.04 %), rutin recovery (96.25 to 96.60 %) and purities of rutin (71.18 to 72.03 %) were observed along 3 extraction cycles related to recycling DESs. These results indicated that the reused DESs did not have any significant effect on extraction efficiency.

**Table 3:** Extraction yields, recovery, and purity of rutin using recovery DESs along 3 extraction cycles relating to recycling solvent.

Cycle	Extraction yields	Recovery yields of rutin	Purity of rutin
1	26.02 ± 0.64	96.60 ± 0.37	71.18 ± 0.47
2	26.04 ± 0.35	96.56 ± 0.33	72.03 ± 0.71
3	25.57 ± 0.38	96.25 ± 0.61	71.24 ± 0.62

## CRediT authorship contribution statement

**Nhan Trong Le:** Conceptualization, Methodology, Investigation, Writing – original draft. **Trieu Phat Dac Nguyen:** Methodology, Investigation, Writing – original draft. **Duc Viet Ho:** Methodology, Investigation, Writing – review & editing. **Huong Thanh Phung:** Methodology, Investigation, Writing – review & editing. **Hoai Thi Nguyen:** Conceptualization, Writing – review & editing, Supervision.

## Declaration of Competing Interest

The authors declare that they have no known competing financial interests or personal relationships that could have appeared to influence the work reported in this paper.
